# Selenium Supplementation in Pregnancy-Maternal and Newborn Outcomes

**DOI:** 10.1155/2022/4715965

**Published:** 2022-05-04

**Authors:** Koushik Biswas, James McLay, Fiona M. Campbell

**Affiliations:** ^1^Rowett Institute, School of Medicine, Medical Sciences and Nutrition, University of Aberdeen, Foresterhill, Aberdeen, AB25 2ZD, UK; ^2^Regional Institute of Ophthalmology, Medical College Hospital, 88 College Street, Kolkata 700073, India; ^3^Division of Applied Health Sciences, University of Aberdeen, Royal Aberdeen Children's Hospital, Aberdeen, AB24 3FX, UK

## Abstract

**Background:**

Several studies have suggested that increased oxidative stress during pregnancy may be associated with adverse maternal and foetal outcomes. As selenium is an essential mineral with an antioxidant role, our aim was to perform a systematic review of the existing literature reporting the effects of selenium supplementation during pregnancy on maternal and neonatal outcomes.

**Materials and Methods:**

Six electronic databases (Medline, Embase, Cochrane Library, Web of Science, Scopus, and PubMed) were searched for studies reporting the effects of selenium supplementation during pregnancy and the postpartum period on maternal and neonatal outcomes. Only randomised controlled trials on human subjects reported in English and published up to October 2021 were included. Quality assessments were conducted using the modified Downs and Black quality assessment tool. Data were extracted using a narrative synthesis.

**Results:**

Twenty-two articles were included in our systematic review (seventeen reported on maternal outcomes, two on newborn outcomes, and three on both). Maternal studies reported the effects of selenium supplementation in the prevention of thyroid dysfunction, gestational diabetes, pregnancy-induced hypertension/preeclampsia, oxidative stress, postpartum depression, premature rupture of membranes, intrauterine growth retardation, breastmilk composition, and HIV-positive women. Newborn studies reported the effects of maternal selenium supplementation on foetal oxidation stress, foetal lipid profile, neonatal hyperbilirubinemia, and newborn outcomes in HIV-positive mothers. The majority of studies were inappropriately designed to establish clinical or scientific utility. Of interest, four studies reported that selenium supplementation reduced the incidence of thyroid dysfunction and permanent hypothyroidism during the postpartum period by reducing thyroid peroxidase and thyroglobulin antibody titres.

**Conclusion:**

The evidence supporting selenium supplementation during pregnancy is poor and there is a need for appropriately designed randomised controlled trials before routine use can be recommended.

## 1. Introduction

Pregnancy places significant demands on maternal nutrients due to both the developing foetus and maternal physiological and hormonal changes. It is recognised that deficiencies of essential vitamins and minerals during this period may lead to the occurrence of perinatal complications, foetus necrobiosis, congenital malformation, and impairment of immune system function in the developing foetus [1–3].

Although current guidelines only advocate iron and folic acid supplementation during pregnancy, there is an increasing interest in the role which other micronutrients may play [1, 4–7]. Selenium (Se), a micronutrient with antioxidant properties, is essential for the synthesis and function of various selenoenzymes such as glutathione peroxidases, selenoproteins P, and thioredoxin reductase, which play important roles in antioxidant defence and limiting oxidative damage [8–12]. Available evidence suggests that Se plasma concentration and glutathione peroxidase activity decrease during pregnancy, due in part to the increasing erythrocyte mass in the developing foetus [13–16].

During pregnancy both maternal and foetal oxygen demand increase, which enhances formation of reactive oxygen species and lipid peroxidation products [15, 17]. It has been previously reported that increased oxidative stress during pregnancy is associated with the occurrence of maternal and foetal/neonatal diseases and adverse outcomes such as miscarriage, preeclampsia, gestational diabetes, premature rupture of membranes, and intrauterine growth restriction. Therefore, Se could be an important micronutrient during the gestational period [18–28]. Se is found in soils and rocks at varying levels across the world. Soil Se level affects plant Se levels, which influences the levels of Se entering the food chain. Consequently, the geographical variation in soil Se levels has a significant effect on dietary Se intake and status in different populations throughout the world [29].

Se supplementation during pregnancy might reduce maternal oxidative stress and have a favourable outcome on both the mother and the foetus [30]. During pregnancy recommended dietary allowance for Se is 60 *μ*mg (0.76 *μ*mol) Se/day [31]. However currently, there are no published systematic reviews assessing the beneficial or adverse effects of Se supplementation during pregnancy. This systematic review aims to retrieve the primary literature reporting the effects of Se supplementation during pregnancy and the postnatal period, to determine whether Se supplementation is associated with any beneficial or adverse maternal and neonatal outcomes.

## 2. Methods

### 2.1. Sources

The Medical Literature Analysis and Retrieval System Online (MEDLINE), Excerpta Medica Database (Embase), Web of Science, the Cochrane Library, Scopus, and PubMed were searched from inception until October 2021. Only publications in English were included.

We applied a three-step search strategy. An initial limited search of MEDLINE was undertaken followed by an analysis of the text words contained in the title and abstract of the article and of index terms used to describe the article. A second search was done using all identified keywords and index terms across all six databases. Thirdly, we searched the reference list of all identified articles and reports for any additional studies. The following search string was used:

(Selenium^∗^ OR “Se Supplement^∗^” OR “Selenium supplement^∗^”) AND (Pregnancy^∗^ OR Pregnan^∗^ OR “Maternal health^∗^” OR “Maternal mortality^∗^” OR “Maternal outcomes^∗^” OR Newborn^∗^ OR Neonat^∗^ OR “Neonatal health^∗^” OR “Neonatal mortality^∗^” OR “Neonatal outcome”) AND (“Outcome” OR “Effect” OR “Treatment effect^∗^” OR “Supplementation effect^∗^”)

### 2.2. Study Selection

Randomised placebo-controlled trials including cluster-randomised trials were considered for inclusion. We excluded articles on quasi-randomised trials, cross-over designs, and all other types of research designs.

For maternal outcomes, all studies that reported any maternal health benefits or harmful effects associated with Se supplementation during pregnancy and postnatal period were included. We considered studies regardless of the stage of pregnancy at which supplementation was started and irrespective of supplementation taken previously.

Similarly, for newborn outcomes, all studies that reported any health benefits or harms in newborns (neonates and infants) following Se supplementation during pregnancy were included irrespective of the health status of the mother.

Quality assessments were conducted by two reviewers (K.B. and J.M.) using modified Downs and Black quality assessment tool for randomised controlled trials included in this study [32, 33]. Downs and Black quality assessment tool score gave corresponding quality levels: excellent (26–28), good (20–25), fair (15–19), and poor (≤14) out of a maximum of 28 points. The relevant items on the quality assessment tool checklist were focused aim of the study, randomisation methodology, participant dropout recording, blinding (single/double), baseline characteristics matching between the two groups, whether factors other than treatment were the same for both groups, treatment effect size, the precision of the study, the applicability of the study findings to the general population, and if all clinically important outcomes were considered [32].

A tailored spreadsheet was constructed for data extraction and data synthesis. All studies identified during the database search were assessed for relevance to our review protocol and quality assessed based on information obtained from the title, abstract, and full-text review by two reviewers (K.B and J.M.). If consensus could not be reached, a third reviewer (F.C.) was consulted.

The key data extracted from selected literature were details of the authors; country and year of publication; Se supplementation product and placebo product used; study population; setting; recruitment; randomisation methodology; blinding technique; baseline characteristics of groups; initiation and duration of supplementation; incidence and magnitude of beneficial/adverse effects; statistical methods used for analysis and risk of bias. Data extracted from trials were the generation of allocation sequence, concealment of allocation, outcome measures, and other risks of bias.

Owing to lack of study homogeneity, a meta-analysis was not appropriate; thus, a narrative synthesis of the results was conducted. Wherever available, statistical data were reported. A systematic review protocol was registered with PROSPERO (CDR42020183126). The PRISMA checklist was used to guide the reporting of this systematic review.

## 3. Results

**Figure 1:**
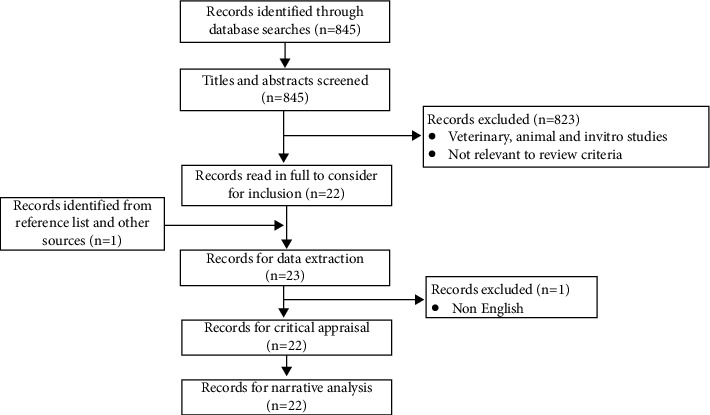
PRISMA (preferred reporting items for systematic reviews and meta-analysis) flowchart.

**Figure 2:**
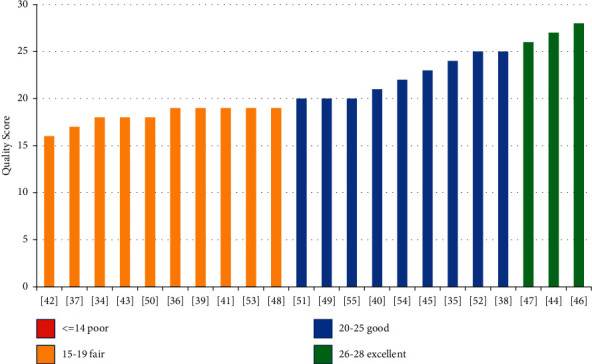
Quality scores of papers included for review (based on Downs and Black assessment tool).

Database searches retrieved a total of 845 citations. After removal of duplicates, title and abstract screening, and full-text screening, 22 articles, meeting inclusion criteria, were critically appraised. These 22 articles were included for data extraction, synthesis, and narrative analysis (Figure 1). Included studies were performed in seven different countries and 18 were primary [34–39, 41–48, 51, 53–55] while four were secondary studies [40, 49, 50, 52]. Ten studies were graded as fair [34, 36, 37, 39, 41–43, 48, 50, 53], nine as good [35, 38, 40, 45, 49, 51, 52, 54, 55], and three as excellent quality [44, 46, 47] (Figure 2). Seventeen studies reported maternal outcomes, two reported neonatal outcomes, and three reported both.

Nine different maternal outcomes were reported. The sample size for studies ranged from 36 to 913. The methods used to identify maternal outcomes were clinical examination, patient questionnaires, laboratory or imaging studies (i.e., haematological, biochemical, gene expression, and ultrasonography), and secondary data analysis. Out of the twenty studies reporting maternal outcomes, three studies did not report significant findings.  Table 1 presents the narrative synthesis of the different maternal outcomes.

Four studies (two classified as fair and two as good; *n* = 45–232) reported on thyroid dysfunction during pregnancy and the postpartum period. Se supplementation did not affect maternal thyroid peroxidase antibody (TPO Ab) or thyroglobulin antibody (Tg Ab) from 12 to 35 weeks of gestation [49, 50] but resulted in a decrease of these antibody titres from 36 weeks' gestation to 6 months' postpartum in a thyroiditis positive population [48]. Postpartum thyroid dysfunction and permanent hypothyroidism were significantly reduced in Se supplemented population compared with controls (28.6 vs. 48.6%, *p* < 0.01; and 11.7 vs. 20.3%, *p* < 0.01) [47].

Three trials reported the effect of Se supplementation on the incidence of pregnancy-induced hypertension (PIH)/preeclampsia (PE). One good quality study conducted on Se deficient women reported that Se supplementation significantly reduced the Odds Ratio for PE/PIH (OR 0·30, 95% CI 0·09, 1·00, *p*=0.049) [53]. A second study of good quality reported that Se supplementation significantly decreased soluble vascular endothelial growth factor receptor 1 (sFLT-1), a biomarker of preeclampsia but did not affect other biomarkers [52]. A third study of fair quality reported that Se supplementation did not significantly decrease the incidence of preeclampsia [51].

Four trials reported the effect of Se supplementation on gestational diabetes mellitus (GDM). One good quality study reported that Se supplementation resulted in a significant reduction in serum insulin levels, fasting plasma glucose, and homeostasis model of assessment- (HOMA-) insulin resistance and a significant increase in quantitative insulin sensitivity check index [38]. A second study, of fair quality, reported that Se supplementation downregulated gene expression of tumour necrosis factor alpha (TNF-*α*) and transforming growth factor beta (TGF-*β*) and upregulated gene expression of VEGF in lymphocytes of patients with GDM [39]. A third study, of good quality, did not find any significant difference in the adiponectin level (which is inversely related to insulin resistance) with Se supplementation [40]. A fourth trial, of fair quality, reported that Se supplementation resulted in upregulation of peroxisome proliferator-activated receptor-*γ* and glucose transporter 1 in lymphocytes of patients with GDM but did not correlate this with any clinical utility observed in the patients [41].

Two small trials, of fair quality, reported that Se supplementation increased breast milk Se concentration but did not change breast milk glutathione peroxidase activity [42,43], increased polyunsaturated fatty acids (*p*=0.02), especially linoleic acid (*p*=0.02), and decreased saturated fatty acids concentration of breast milk (*p*=0.04) [43].

Three trials were conducted on HIV-positive pregnant women. One excellent quality study reported that Se supplementation had no significant effect on maternal CD4 cell count, viral load, and maternal mortality (RR = 1.02; 95% CI = 0.51, 2.04; *p*=0.96) [44]. The second study, of good quality, reported that Se supplementation increased detectable HIV-1 RNA in breast milk (36.4% vs. 27.5%) [45]. The third trial, of excellent quality, reported that Se supplementation significantly lowered the risk of preterm delivery (relative risk 0.32, 95% confidence interval 0.11–0.96) compared to placebo. But it did not affect HIV-disease progression in pregnant women, as evidenced by no significant changes in CD4+ cell count [46].

A fair quality trial reported that Se supplementation during pregnancy effectively reduced the incidence of premature rupture of membranes when compared to placebo (13.1% vs. 34.4%, *p* < 0.01) [34]. Another small study of fair quality reported that the mean Edinburgh Postnatal Depression Scale (EPDS) score in Se supplemented group was significantly lower than the control group (8.8 + 5.1 vs. 10.7 + 4.4, *p* < 0.05); thus it could help in reducing postpartum depression [36].

A small fair quality trial reported that, after 10 weeks of Se supplementation, a higher percentage of women in the Se group had a pulsatility index (PI) of <1.45 (*p*=0.002) compared to those in the placebo group, suggesting it could help in reducing intrauterine growth retardation. However, newborn birth weight was not compared in either the treated or the control groups [35]. Another fair quality study reported that Se supplementation reduced oxidative stress associated with pregnancy, as demonstrated by PAB assay [167.3 (135.4–221) vs. 221.0 (162.0–223.3), *p* < 0.05]. However, this study lacked any clinical utility [37].

Five trials reported the effect of Se supplementation on neonatal outcomes (Table 2). An excellent quality trial reported that Se supplementation in HIV-positive mothers reduced risk of low birth weight babies [relative risk (RR) = 0.71; 95% CI: 0.49, 1.05; *p*=0.09*p*=0.09) and child mortality after 6 weeks (RR = 0.43; 95% CI = 0.19, 0.99; *p*=0.048) but increased the risk of foetal death (RR = 1.58; 95% CI = 0.95, 2.63; *p*=0.08) [44]. Another excellent trial reported that Se supplementation resulted in a nonsignificant reduction in the risk of delivering low birth weight babies at term pregnancy (RR 0.24, 95% CI 0.05–1.19) [46]. A fair quality trial reported that maternal Se supplementation increased cord blood Se (106.3 ± 18.2 vs. 101.9 ± 15.9, *p*=0.29) and increased foetal PAB [37.2(26.1–121.0) vs. 30.8(24.0–45.5), *p*=0.19] [54]. Another fair quality trial reported that maternal Se supplementation increased cord blood triglyceride level (56 vs. 38.5 mg/dl) (*p* < 0.001) [53]. There was no effect of Se supplementation on the foetal sex, gestational age at birth, birth weight, birth length, head circumference, Apgar scores at 1 and 5 minutes, and newborn mortality and morbidity [54, 55]. The fifth study, of fair quality, reported that selenium supplementation in GDM patients significantly decreased the incidence of hyperbilirubinemia (5.6% vs. 33.3%, *p*=0.03) and hospitalization (5.6% vs. 33.3%, *p*=0.03) in newborn infants [41].

## 4. Discussion

The majority of studies included in our review reported Se supplementation in pregnancy to be of benefit, reducing PE/PIH, GDM, IUGR, PROM, postpartum depression, and postpartum thyroid dysfunction. Se supplementation is also reported to affect breast milk composition, foetal lipid profile, and foetal bilirubin level and has mixed outcomes in HIV-positive mothers and their newborns.

It is reported that oxidative stress increases during pregnancy [37] and this increased stress is associated with many pregnancy-related illnesses. The blood level of Se decreases during pregnancy mainly due to haemodilution and transport to the developing foetus. Thus, Se, which has antioxidant properties, may be a highly useful supplement during gestation to overcome this increased oxidative stress.

We identified a small number of randomised controlled trials focusing on maternal and newborn outcomes following Se supplementation. Although the majority of available studies were graded as fair and good, results were contradictory and there were significant methodological issues in the published reports which limited interpretability and generalisability.

The main finding in this review is the evidence that Se supplementation helps to reduce postpartum thyroid dysfunction. A well-designed study by Negro et al. [47], graded as excellent, reported that Se supplementation decreased TPO Ab in the postpartum period and reduced the incidence of postpartum thyroid dysfunction and permanent hypothyroidism. Mantovani et al. [48] in a small study (*n* = 45) reported that Se supplementation decreased Tg Ab from 36 weeks' pregnancy to 6 months' postpartum period in thyroiditis positive women. Both these studies suggest that Se supplementation may help reduce postpartum thyroid dysfunction; however further research is needed to validate these findings and determine the therapeutic Se dose for such patients.

Another important finding of our review is the evidence for Se supplementation in HIV-positive pregnant women. A well-designed trial by Kupka et al. [44] reported that supplementation in HIV-positive mothers reduced the risk of low birth weight babies and child mortality after 6 weeks of birth but increased the risk of foetal death. Another well-designed trial by Okunade et al. [46] reported that selenium supplementation in HIV-positive mothers significantly reduced the risk of preterm delivery. A third study on HIV-positive mothers by Sudfeld et al. [45] reported that Se supplement increased detectable HIV-1 RNA in breast milk among women who did not take antiretroviral drugs and thus Se could increase the risk of mother-to-child transmission of HIV. Hence, Se supplementation may better be avoided in HIV-positive mothers until a robust advantage is evident.

The main advantage of our study is that we included only single or double-blind randomised controlled trials for review; we did not rely on observational data from other types of study. Our study was not without limitations. Out of the 22 studies, four were secondary studies [40, 49, 50, 52] which were originally designed to study other aspects of Se supplementation. Only two primary studies measured Se status of participants at the baseline [48, 51]. Only five studies had a power calculation to determine the sample size [38, 41, 44, 46, 47]. Only three studies included in our review were of excellent quality. The dose and composition of Se supplement were not uniform in all the studies. Adverse events from Se supplement use were underreported in these studies. Six studies conducted in Iran [34, 36, 37, 51, 54, 55] and five studies conducted in the UK [40, 49–52] were on the same population cohort, respectively.

## 5. Conclusion

Available evidence confirms the effect of Se supplementation on maternal or newborn outcomes is understudied and currently unknown. The evidence-based use of nutritional supplementation in pregnancy must be backed by robust appropriately designed and powered clinical trials. Currently, there is insufficient information to recommend the safe use of Se supplementation during pregnancy and the postnatal period. The safe dosage of Se supplement in pregnancy needs to be determined.

## Figures and Tables

**Table 1:** Reported maternal outcomes on Se supplementation.

Clinical condition	Result of Se supplementation	Reference/Country/Sample size (*n*)/Quality
Premature rupture of membranes (PROM)	Se supplementation during pregnancy effectively reduced the incidence of PROM when compared to placebo (13.1% vs. 34.4%, *p* < 0.01)	[34]/Iran/*n* = 125/fair
Intrauterine growth retardation (IUGR)	After 10 weeks of Se supplementation, a higher percentage of women in the Se group had pulsatility index (PI) of <1.45) (*p*=0.002) than of those in the placebo group. However, no comparison was made of the birth weight of babies in both groups.	[35]/Iran/*n* = 60/good
Postpartum depression	The mean Edinburgh Postnatal Depression Scale (EPDS) score in the selenium supplemented group was significantly lower than the control group (8.8 + 5.1 vs. 10.7 + 4.4, *p* < 0.05)	[36]/Iran/*n* = 85/fair
Oxidative stress	Se supplementation reduced oxidative stress associated with pregnancy, as demonstrated by the PAB assay (167.3 (135.4–221) vs. 221.0 (162.0–223.3), *p* < 0.05)	[37]/Iran/*n* = 125/fair
Gestational diabetes	Se supplementation, compared with placebo, resulted in a significant reduction in fasting plasma glucose, serum insulin levels, and homeostasis model of assessment- (HOMA-) insulin resistance and a significant increase in quantitative insulin sensitivity check index	[38]/Iran/*n* = 70/good
	Se supplementation downregulated gene expression of tumour necrosis factor alpha (TNF-*α*) and transforming growth factor beta (TGF-*β*) and upregulated gene expression of VEGF in lymphocytes of patients with GDM	[39]/Iran/*n* = 40/fair
	Se supplementation did not cause any significant difference in the adiponectin level (which is inversely related to insulin resistance) from 12 to 35 weeks of gestation (*p*=0.938)	[40]/UK/*n* = 230/good
	Se supplementation resulted in upregulation of peroxisome proliferator-activated receptor-*γ* (*p*=0.03) and glucose transporter 1 (*p*=0.01) in lymphocytes of patients with GDM.	[41]/Iran/*n* = 36/fair
Breastmilk	Se supplementation increased breastmilk selenium level but did not significantly increase breastmilk glutathione peroxidase activity	[42]/China/*n* = 20/fair
	Se supplementation increased breastmilk Se concentration (*p*=0.003), did not change breastmilk glutathione peroxidase activity, significantly increased the concentration of polyunsaturated fatty acids (*p*=0.02), mainly linoleic acid (*p*=0.02), and decreased the concentration of saturated fatty acids in breast milk (*p*=0.04)	[43]/New Zealand/*n* = 22/fair
Human immunodeficiency virus (HIV) positive women	No significant effect on maternal CD4 cell count, viral load, and maternal mortality (RR = 1.02; 95% CI = 0.51, 2.04; *p*=0.96)	[44]/Tanzania/*n* = 913/excellent
	The proportion of women with detectable HIV-1 RNA in breast milk increased in Se supplemented (36.4%) than placebo (27.5%) group. The effect was more in primiparas.	[45]/Tanzania/*n* = 420/good
Human immunodeficiency virus (HIV) positive women	Se supplementation significantly lowered risk of preterm delivery (relative risk (RR) 0.32, 95% confidence interval (CI) 0.11–0.96) compared to placebo. Se supplementation caused no effect on HIV-disease progression in pregnant women.	[46]/Nigeria/*n* = 180/excellent
Thyroid disorder	Se supplementation lowered TPO Ab titres during the postpartum period compared to controls (323.2 ± 44 vs. 621.1 ± 80 kIU/litre) (*p* < 0.01). Postpartum thyroid dysfunction and permanent hypothyroidism were significantly lower in selenium supplemented population compared with controls (28.6 vs. 48.6%, *p* < 0.01; and 11.7 vs. 20.3%, *p* < 0.01).	[47]/Italy/*n* = 232/excellent
	From 36 weeks' gestation to 6 months' postpartum, Se supplementation decreased Tg Ab [19.86 (11.59–52.60) IU/ml; *p* < 0.01] in a thyroiditis positive population but it increased in the control group (151.03 ± 182.9 IU/ml; *p* < 0.01). TPO Ab also decreased on Se supplementation (255.00 (79.00–292.00) IU/ml; *p* < 0.01) but increased in the control group (441.28 ± 512.18 IU/ml; *p* < 0.01) during the same period.	[48]/Italy/*n* = 45/fair
Thyroid disorder	Low-dose Se supplementation in pregnant women with mild-to-moderate deficiency had no effect on TPO Ab concentration from 12 to 35 weeks of gestation but tended to change thyroid function in TPO Ab + ve women in late gestation (35 weeks): reduced TSH (2.10 (1.83, 2.38) vs. 2.50 (2.24, 2.79) mU/l, *p*=0.05*p*=0.05), reduced FT4 (10.54 (9.83,11.25) vs. 11.67 (11.03 vs. 12.31) pmol/l, *p*=0.029*p*=0.029)	[49]/UK/*n* = 229/good
	Se supplementation did not affect TPO Ab or TSH level in pregnant women. During late gestation (35 weeks), serum FT4 levels were lower in the selenium-treated women compared to controls (10.54 vs. 10.82 pmol/l, *p*=0.029).	[50]/UK/*n* = 230/fair
Pregnancy-induced hypertension (PIH)/preeclampsia (PE)	There was no incidence of preeclampsia in the treated group, but 4.7% (*n* = 3) of women in the control group suffered from preeclampsia (statistically nonsignificant)	[51]/Iran/*n* = 125/good
	Se supplementation significantly lowered the concentration of soluble vascular endothelial growth factor receptor 1 (sFlt-1), which is a biomarker of preeclampsia, among the Se deficient women. However, the difference in the concentration of other biomarkers was not significant.	[52]/UK/*n* = 230/good
Pregnancy-induced hypertension (PIH)/preeclampsia (PE)	Se supplementation in a Se deficient UK population significantly reduced the odds ratio for PE/PIH (OR 0·30, 95% CI 0·09, 1·00, *p*=0.049)	[53]/UK/*n* = 230/fair

**Table 2:** Reported neonatal outcomes on Se supplementation.

Clinical condition	Result of Se supplementation	Reference/Country/Sample size (*n*)/Quality
Neonatal oxidative stress	Se supplementation increased foetal cord blood selenium levels (106.3 ± 18.2 vs. 101.9 ± 15.9, *p*=0.29) and increased foetal PAB (37.2(26.1–121.0) vs. 30.8(24.0–45.5), *p*=0.19). But there was no effect on foetal birth weight, gestational age at birth, Apgar score at 1 and 5 minutes, newborn mortality, and morbidity.	[54]/Iran/*n* = 125/good
Cord blood Se and lipid profile	Se supplementation during pregnancy did not significantly change the cord blood selenium (106.3 vs. 101.9 *μ*g/L), total cholesterol (96.7 vs. 79.6 mg/dl), LDL-C (58 vs. 45.1 mg/dl), and HDL-C (23 vs. 20.2 mg/dl) levels but increased the serum triglyceride level (56 vs. 38.5 mg/dl) (*p* < 0.001). There was no effect of Se supplementation on the foetal sex, gestational age at birth, birth weight, birth length, head circumference, and Apgar scores at 1 and 5 minutes.	[55]/Iran/*n* = 66/good
HIV	Maternal Se supplementation reduced risk of low birth weight babies (relative risk (RR) = 0.71; 95% CI: 0.49, 1.05; *p*=0.09) and risk of child mortality after 6 weeks (RR = 0.43; 95% CI = 0.19, 0.99; *p*=0.048), but it increased risk of foetal death (RR = 1.58; 95% CI = 0.95,2.63; *p*=0.08). There was no effect on risk of prematurity or small-for-gestational age birth.	[44]/Tanzania/*n* = 913/excellent
	Se supplementation resulted in a nonsignificant reduction in the risk of delivering low birth weight babies at term pregnancy (RR 0.24, 95% CI 0.05–1.19).	[46]/Nigeria/*n* = 180/excellent
Newborn Hyperbilirubinemia	Selenium supplementation in GDM patients significantly decreased incidence of newborns' hyperbilirubinemia (5.6% vs. 33.3%, *p*=0.03) and newborns' hospitalization (5.6% vs. 33.3%, *p*=0.03).	[41]/Iran/*n* = 36/fair

## Data Availability

The data used to support the findings of this study are included within the article.
